# Identification and validation of a signature involving voltage-gated chloride ion channel genes for prediction of prostate cancer recurrence

**DOI:** 10.3389/fendo.2022.1001634

**Published:** 2022-09-30

**Authors:** Yong Luo, Xiaopeng Liu, Xiaoxiao Li, Weide Zhong, Jingbo Lin, Qingbiao Chen

**Affiliations:** ^1^ Department of Urology, The Second People’s Hospital of Foshan, Affiliated Foshan Hospital of Southern Medical University, Foshan, China; ^2^ Department of Science and Teaching, The Second People’s Hospital of Foshan, Affiliated Foshan Hospital of Southern Medical University, Foshan, China; ^3^ Department of Nursing Administration, the Second People’s Hospital of Foshan, Affiliated Foshan Hospital of Southern Medical University, Foshan, China; ^4^ Department of Urology, Guangdong Key Laboratory of Clinical Molecular Medicine and Diagnostics, Guangzhou First People’s Hospital, School of Medicine, South China University of Technology, Guangzhou, China; ^5^ Urology Key Laboratory of Guangdong Province, The First Affiliated Hospital of Guangzhou Medical University, Guangzhou Medical University, Guangzhou, China; ^6^ Guangdong Provincial Institute of Nephrology, Nanfang Hospital, Southern Medical University, Guangzhou, China; ^7^ State Key Laboratory of Quality Research in Chinese Medicines, Macau University of Science and Technology, Macau, Macau SAR, China

**Keywords:** prostate cancer, recurrence, CLCN2, CLCN6, cell experiment

## Abstract

Voltage-gated chloride ion channels (CLCs) are transmembrane proteins that maintain chloride ion homeostasis in various cells. Accumulating studies indicated CLCs were related to cell growth, proliferation, and cell cycle. Nevertheless, the role of CLCs in prostate cancer (PCa) has not been systematically profiled. The purpose of this study was to investigate the expression profiles and biofunctions of CLCs genes, and construct a novel risk signature to predict biochemical recurrence (BCR) of PCa patients. We identified five differentially expressed CLCs genes in our cohort and then constructed a signature composed of CLCN2 and CLCN6 through Lasso-Cox regression analysis in the training cohort from the Cancer Genome Atlas (TCGA). The testing and entire cohorts from TCGA and the GSE21034 from the Gene Expression Omnibus (GEO) were used as internal and independent external validation datasets. This signature could divide PCa patients into the high and low risk groups with different prognoses, was apparently correlated with clinical features, and was an independent excellent prognostic indicator. Enrichment analysis indicated our signature was primarily concentrated in cellular process and metabolic process. The expression patterns of CLCN2 and CLCN6 were detected in our own cohort based immunohistochemistry staining, and we found CLCN2 and CLCN6 were highly expressed in PCa tissues compared with benign tissues and positively associated with higher Gleason score and shorter BCR-free time. Functional experiments revealed that CLCN2 and CLCN6 downregulation inhibited cell proliferation, colony formation, invasion, and migration, but prolonged cell cycle and promoted apoptosis. Furthermore, Seahorse assay showed that silencing CLCN2 or CLCN6 exerted potential inhibitory effects on energy metabolism in PCa. Collectively, our signature could provide a novel and robust strategy for the prognostic evaluation and improve treatment decision making for PCa patients.

## Introduction

Prostate cancer (PCa) has become one of the world’s most frequently diagnosed aggressive malignant tumors in men urogenital system that not only poses a grave danger to men’s health but also simultaneously imparts economic loss to society and the family (1). Prostate-specific antigen (PSA), a routinely and extensively utilized PCa detection test, has been widespread used for PCa prognosis (2). Nevertheless, approximately 23% of PCa patients who received radical prostatectomy (RP) and adjuvant radiation therapy would suffer from PSA recurrence within 2 or 3 years (3). Biochemical recurrence (BCR) could contribute to an increased risk of advanced castration-resistant PCa (CRPC), progressing into clinical relapses and distant metastases that ultimately lead to prostate cancer-specific death (4). Gene expression alterations or mutations that are extraordinarily related to BCR has great importance for the predictive recurrence of PCa patients (5). Thus, early identification of effective biomarkers for PCa patients with high BCR risk is urgently needed.

Ion channels are pore-forming membrane proteins allowing ions to pass through channel pores. According to the selectivity of ion channels, they can be classified as chloride channels, potassium channels, sodium channels, etc. Chloride channels are channel proteins present in the cell membrane that are highly selective for chloride ions. It has been shown that chloride ions were involved in various physiological and pathophysiological processes such as cell proliferation, cell cycle regulation, apoptosis, cell volume regulation, and pH regulation (6–8). Voltage-gated chloride ion channels (CLCs), belonging to the mammalian chloride channels family, are a series of transmembrane proteins that maintain chloride ion homeostasis in various cells (9, 10). Alterations of cell volume played a vital role in both cell proliferation and cell apoptosis. The signals for cell proliferation required an increase of cell volume at some stage, while cell apoptosis was paralleled by cell shrinkage (11). Chloride ion transporters were expressed in the cell membrane, which could modify ion activity, mediate osmolyte flux, and alter cell volume. The chloride channels could release HCO- 3, contributing to cytosolic acidification, which suppressed cell proliferation and promoted cell apoptosis (8).

Sustaining proliferative signaling, enabling replicative immortality, evading growth suppressors, resisting cell death, and derelugulating cellular energetics were the hallmarks of cancer (12, 13). Ion channels and transporters as a novel class of membrane proteins were aberrantly expressed in human cancers and were promising cancer biomarkers (14). CLCN2, a biomarker of esophageal squamous cell carcinoma, regulated tumor progression and predicted survival outcomes *via* affecting IFN signaling (15). Xu et al. pointed out that CLCN3 was essential for cell cycle progression and cell proliferation, which could represent a potential therapeutic target in nasopharyngeal carcinoma cells (6). It was revealed that CLCN4 overexpression promoted cell migration, invasion, and metastases, and CLCN4 was a novel driver in colon cancer (16). CLCN5 downregulation could promote cell apoptosis by activating mitochondria-dependent apoptotic pathway in osteosarcoma (17). Additionally, previous studies revealed that dysregulation of metabolism was linked to the progression and carcinogenesis of PCa (18, 19). The pathological and physiological functions of mitochondria significantly relied on the properties and regulation of ion channels on the mitochondrial membrane. Pan et al. reported that an accumulation of glucose concentration inhibited keratinocyte migration by way of downregulation of CLCN2, indicating CLCN2 had a pronounced implication in wound epithelialization (20). besides, the deceleration of CLCN2 common gating to maintain the electrical stability of neurons was triggered by ATP, which also altered CLCN2 surface expression (21). ATP was the main form of biological energy, most of which was derived from mitochondrial metabolism (22). Mitochondria was a center for oxidative metabolism and made a response to cellular stress such as cell proliferation, invasion, apoptosis, and metastasis (23, 24). Nevertheless, few research was conducted on the relationship between the expression patterns of CLCs and the progression and prognosis of PCa at the cellular or metabolic levels.

In this study, we set out to construct and validate a novel signature based on CLCs related genes that could predict the BCR of PCa. Our signature comprising of CLCN2 and CLCN6 was greatly related to various clinicopathological characteristics and could be an independent excellent prognostic factor. Enrichment analysis indicated that the signature was extraordinarily associated with cellular process and metabolic process. Furthermore, we investigated the clinical significance of CLCN2 and CLCN6 expression along with their biological functions in PCa based on abundant experiments analysis. What we worked out in this present study provided new insights of CLCs in the progression, prognosis, and personalized therapeutic targets for PCa.

## Materials and methods

### Data acquisition and preprocessing

Human prostate tissue microarrays (TMA; Alenabio, China, PR803d), containing 62 prostate adenocarcinoma tissues, 4 prostate leiomyosarcoma tissues, 6 prostate hyperplasia tissues, and 8 normal tissues, were used for immunohistochemical staining (IHC). Prostate leiomyosarcoma tissues and patients with preoperative chemotherapy or radiotherapy were all excluded from this study. Another two cohorts consisting of gene expression profiles and clinical information of 499 TCGA-PRAD patients along with 52 paired benign samples and GSE21034 cohort including 140 PCa tissues were retrieved from the Cancer Genome Atlas (TCGA: https://portal.gdc.cancer.gov/) and Gene Expression Omnibus (GEO: https://www.ncbi.nlm.nih.gov/geo/), respectively. Detail clinical information of TMA comprising of age, Gleason score, and tumor stage was summarized in **Table 1**. The exclusion criteria of TCGA-PRAD and GSE21034 cohorts was PCa patients without either recorded BCR time or BCR outcomes. The definition of BCR was successive PSA levels > 0.2 ng/mL for twice along with an elevated trend after RP. Additionally, we introduced BPH-1 (benign prostate cell line) and PC3 (PCa cell line) to screen out differentially expressed CLCs. The total mRNA was isolated and extracted for RNA-sequencing by the Sinotech Genomics (China). The RNA-sequencing data has been uploaded to the GEO database (GSE210205).

**Table 1:** The relation of CLCN2 and CLCN6 expression to relevant clinical features of PCa patients in our cohort.

Clinical features	CLCN2			CLCN6		
	Case	$\bar{X}\pm \text{s}$	P	Case	$\bar{X}\pm \text{s}$	P
**Expression**						
Benign	14	3.86±2.14	**0.035**	13	3.31±1.84	**0.001**
Cancer	59^#^	6.05±3.67		61^##^	6.77±3.12	
**Age(years)**						
≤60	6	6.17±4.12	0.936	7	5.86±3.93	0.414
>60	53	6.04±3.66		54	6.89±3.02	
**Gleason score** ^Δ^						
≤7	27	5.15±3.46	**0.047**	28	6.18±2.76	**0.041**
>7	31	7.03±3.56		32	7.13±3.23	
**Tumor stage**						
I-II	28	6.29±3.74	0.506	38	6.74±3.07	0.915
III-IV	21	5.62±3.58		23	6.83±3.26	

#3 prostate adenocarcinoma tissues dropped off, ## 1 prostate adenocarcinoma tissue dropped off, Δ 1 is lack of relative information of Gleason score. Bold values indicate statistical significance (p<0.05).

### Development and evaluation of a CLCs signature

The CLCs family, including CLCN1, CLCN2, CLCN3, CLCN4, CLCN5, CLCN6, CLCN7, CLCNKA, and CLCNKB, was conducted to screen out differentially expressed CLCs based on RNA-sequencing, with the a criterion of *P* value< 0.05. The *caret* R software was applied to randomly divide the entire TCGA-PRAD dataset into a training cohort and a testing cohort. The training cohort was appointed to build the signature, which was validated in the testing cohort, entire cohort, and GSE21034 cohort. Then, we performed Cox regression analysis to assess the correlation between the expression level of each gene and the prognosis. Next, we obtained candidate genes to develop a CLCs signature by least absolute shrinkage and selection operator (Lasso) with 10-fold cross-validation. Finally, we retained two genes and coefficients with penalty parameter (λ) determined by the minimum criterion. The formula to calculate the risk score was as follows:


$$\text{RiskScore}=\sum_{i}^{\lambda }\beta \text{iSi}$$

The median value of risk score classified PCa patients into the high-risk and low-risk groups. The time-dependent receiver operating characteristic (ROC) was used to assess the area under curve (AUC) and the accuracy of prognostic prediction. Univariate and multivariate Cox regression analyses were utilized to confirm the independence of the signature. The R packages employed here included *timeROC*, *survival*, and *survminer*. Additionally, we performed Wilcoxon signed-rank test and chi-square test to investigate the relation of the signature to clinicopathological characteristics of PCa patients through scatter diagram and strip chart respectively.

### Distribution analysis, establishment of a nomogram, and enrichment analysis

t-SNE and PCA analyses were performed to distinguish the distribution patterns of every individual belonging to two subgroups *via Rtsne*, *ggplot2*, and *scatterplot3d* R packages. A nomogram was conducted in terms of BCR-free survival and the calibration curve was applied to assess the predictive accuracy based on *rms* R package. To determine the cancer-related pathways correlated to the signature, we screened out a series of differentially expressed genes (DEGs) between two subgroups to investigate the biological processes and potential molecular mechanisms. Gene Ontology (GO) and Kyoto Encyclopedia of Genes and Genomes (KEGG) analyses were analyzed by *clusterProfiler* R package. Gene set enrichment analysis (GSEA) was utilized to explore the enriched biological process, cellular component, molecular function, and signaling pathway.

### Immunohistochemistry

The subcellular localization and expression levels of CLCN2 (Bioss, China, bs-6470R) and CLCN6 (Sigma, USA, HPA032097) in clinical PCa tissues and benign prostate specimens were detected by IHC according to the protocol of our previous study (25). The number and percentage of positively stained cells were calculated with reference to our previous study (25). The CLCN2 and CLCN6 protein levels were assessed by staining regions classified as follows: 0, 0-5%; 1, 6-25%; 2, 26-50%; 3, 51-75%; 4, 76-100% and intensity of staining categorized as 0 (negative), 1 (weak), 2 (moderate), and 3 (strong). The final immunoreactivity score (IRS) in each specimen was obtained by multiplying the percentage and intensity score.

### Cell culture and transfection

Two non-androgen dependent PCa DU145 and PC3 cell lines were derived from American Type Culture Collection (ATCC, USA) and were cultured accordingly in DMEM (Gibco, USA) supplemented with 10% fetal bovine serum (FBS Gibco, USA), 100 U/mL penicillin, and 0.1mg/mL streptomycin (Gibco, USA). PCa cell lines were incubated in a humidified atmosphere supplemented with 5% CO_2_ at 37°C. Transfection of both DU145 and PC3 cell lines using siRNAs or control siRNA was carried out according to the manufacturer’s instructions (Genepharma, China, **Table 2**) (26). The expression levels of CLCN2 and CLCN6 protein in PCa DU145 and PC3 cell lines with transfection of siRNA reagent or control siRNA were detected by Western blot (WB) assay based on our previous report (27).

**Table 2:** siRNA sequences of CLCN2 and CLCN6.

	Sense	Antisense
CLCN2-siRNA1	GCUUGAACACCAGCAUCUUTT	AAGAUGCUGGUGUUCAAGCTT
CLCN2-siRNA2	GCAAGUUCCUCUCCCUCUUTT	AAGAGGGAGAGGAACUUGCTT
CLCN2-siRNA3	GCCUUUGCUGUCAUUGGUATT	UACCAAUGACAGCAAAGGCTT
CLCN6-siRNA1	CCGGGAUACCCGAGGUCAATT	UUGACCUCGGGUAUCCCGGTT
CLCN6-siRNA2	GCUGAACUUUGGCGAGUUUTT	AAACUCGCCAAAGUUCAGCTT
CLCN6-siRNA3	CCAUCAGCCUCACGGUCAUTT	AUGACCGUGAGGCUGAUGGTT

### Cell proliferation, colony formation, migration, and invasion assays

Based on the protocol of our previous study (28), the effects of CLCN2 and CLCN6 on cell proliferation, colony formation, migration, and invasion in DU145 and PC3 cells transfected with CLCN2/CLCN6-siRNA reagent or control siRNA were detected by CCK-8 assay, colony formation assay, wound-healing assay, and transwell assay.

Cell proliferation was detected by cell counting kit-8 (CCK-8, Meilunbio, China) and colony formation. Cell suspensions containing approximately 5000 cells/100 μL were seeded into a 96-well plate. Then 10 μL CCK-8 solution was added to each well after culturing for 4 - 72h. The absorbance was measured at 450 nm by a microplate reader (Bio-Rad, USA) when the cells were incubated for two hours. For colony formation assay, 500 cells of per well were seeded in the six-well plate with the corresponding medium for two weeks. Then, 0.1% crystal violet was used to fix and stain the cells at room temperature for an hour. The results were photographed with a full view of each well. In addition, cell suspensions were seeded in the six-well plate and then reached approximately 90% confluence for migration assay. A linear scratch wound on each well was made in monolayers with a 200μL sterile pipette tip. Representative photos through the scratch wound were taken using a microscope camera at 0, 24, and 48 h. For invasion assay, the surface of the upper chamber was previously coated with matrigel (BD Biosciences, USA). Approximately 50000 cells suspended in serum-free DMEM medium were seeded in the upper compartment of the chamber, while the lower chamber contained the normal medium as an attractant. After 24h incubation for DU145 and 48h incubation for PC3, the membrane was removed, fixed with 4% paraformaldehyde, and stained with 0.1% crystal violet at room temperature for one hour. Representative photos of the invading cells were taken using a microscope and the number of them were calculated in five randomly-selected fields. All of the experiments above were repeated more than three times.

### Cell cycle assay and apoptosis assay

Cell suspensions were seeded in the six-well plate at a confluence of approximately 70-80% per well for cell cycle assay. After fixed in 70% ethyl alcohol at -4 °C overnight, the cells were resuspended in PBS containing 50 μg/mL PI, 100 μg/mL RNaseA, and 0.5% Triton X-100, and incubated in a dark box for 30 min at 4 °C later. Flow cytometer (BD Biosciences, USA) was conducted to analyze the cell cycle. For the apoptosis assay, the cells were seeded, collected, washed, and stained with binding buffer, Annexin V, and 7-AAD, and then performed to be analyzed by the FACScan flow cytometer (BD Biosciences, USA). Flowjo software was used for further analysis. All of the experiments above were conducted in more than three times.

### Seahorse assay

Mitochondrial function was detected by measuring the oxygen consumption rate (OCR) using XF Cell Mito Stress Test Kit (Agilent Technologies) and glycolytic function was determined by measuring the extracellular acidification rate (ECAR) using XF Glycolysis Stress Test Kit (Agilent Technologies). Briefly, 6000 cells/80μL were seeded in an XF96 cell culture microplate with complete medium for two days. Assay medium was prepared by supplementing Seahorse XF Base Medium with 1 mM glutamine added for ECAR measurements and 1 mM pyruvate, 2 mM glutamine, and 10 mM glucose added for OCR measurements. A sensor cartridge was hydrated in Seahorse XF calibrant at 37 °C in a non-CO_2_ incubator overnight and Seahorse XF96 Analyzer turned on to let it warm up for six hours in advance. Then, the assay medium was used to wash and incubate the cells in a 37°C incubator without CO_2_ for one hour prior to the assay. For the mito stress test, the cells were exposed sequentially to oligomycin (0.5 μM), fluorocarbonyl-cyanide-phenylhydrazone (FCCP, 1 μM), and rotenone/antimycin A (Rot/AA, 0.5 μM). For the glycolysis stress test, the concentration of the compounds was separately changed to glucose (10 mM), oligomycin (1 μM), and 2-deoxy-D-glucose (2-DG, 50 mM). The results were analyzed using the Wave program 2.6.0 (Seahorse Bioscience).

### Statistical analysis

All statistical analyses were performed by R version 4.1.1, GraphPad Prism 8, and SPSS 26. Wilcoxon signed-rank test and chi-square test were used to analyze the statistical significance of different groups. Continuous variables were presented as mean ± standard deviation (SD). *P*< 0.05 was considered statistically significant in all results.

## Results

### Development and evaluation of a CLCs signature

**Figure 1:**
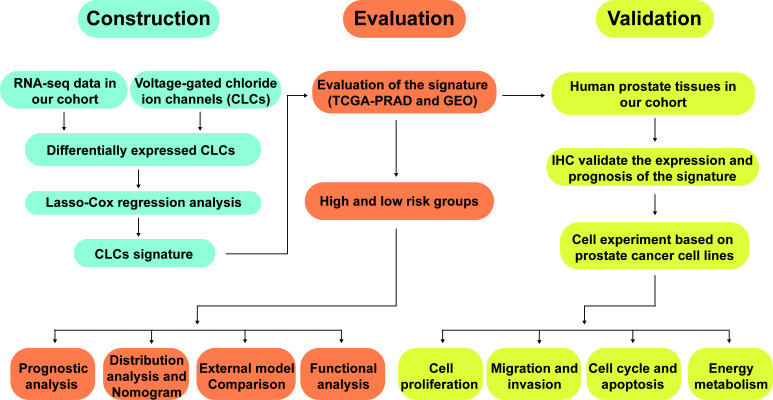
The workflow diagram of this present study.

**Figure 2:**
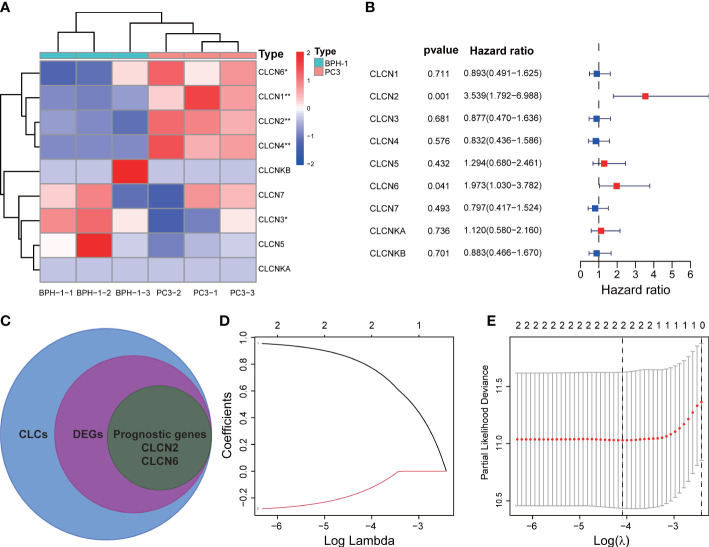
Development of the signature. **(A)** Identification of differentially expressed CLCs by RNA-sequencing. **(B)** Univariate Cox analysis of CLCs based on the TCGA-PRAD cohort. **(C)** Venn diagram of DEGs and prognostic genes. **(D, E)** Identification of a CLCs signature by the Lasso-Cox regression. * p<0.05 and ** p<0.01.

**Figure 3:**
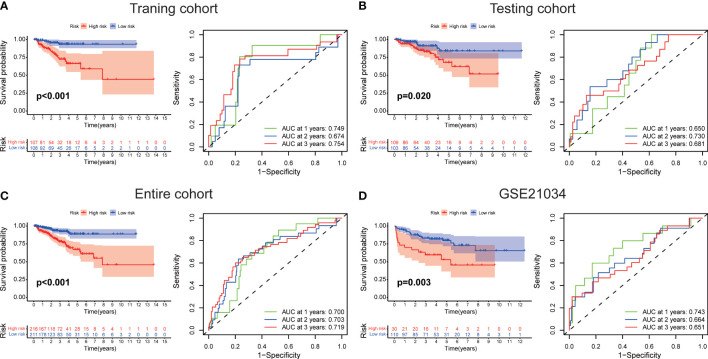
Evaluation of the signature. The BCR-free survival and ROC curves in **(A)** the training TCGA-PRAD, **(B)** the testing TCGA-PRAD, **(C)** entire TCGA-PRAD, and **(D)** GSE21034 cohorts.

**Figure 4:**
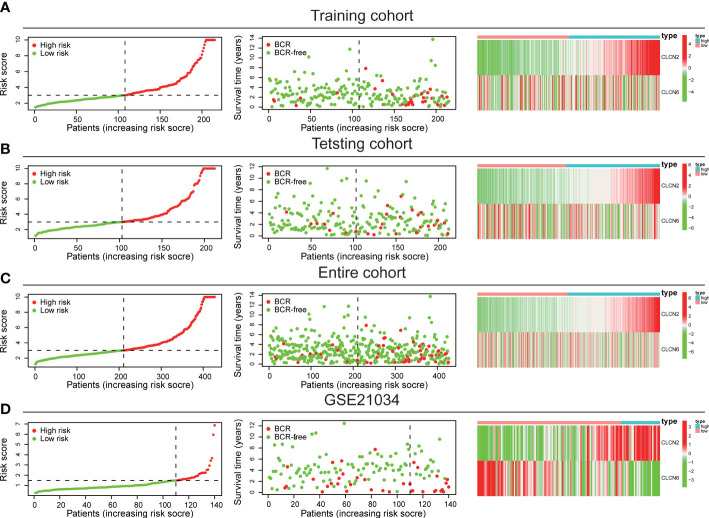
Internal and external validation of the signature. The distribution of risk scores, survival status, and heatmap in **(A)** the training TCGA-PRAD, **(B)** the testing TCGA-PRAD, **(C)** entire TCGA-PRAD, and **(D)** GSE21034 cohorts.

The workflow diagram of this present study, including Construction, Evaluation, and Validation sections, was developed in **Figure 1**. We first obtained five differentially expressed CLCs from CLCs family based on RNA-sequencing of BPH-1 and PC3 (**Figure 2A**). To comprehensively explore the prognostic value of CLCs family, we determined two CLCs from the TCGA-PRAD cohort by the uni-Cox analysis (**Figure 2B**). Venn diagram depicted the screening process of core genes (**Figure 2C**). PCa patients were divided into the training cohort and the testing cohort randomly and equally. We performed Lasso regression analysis in the training cohort and two CLCs (CLCN2 and CLCN6) were used to construct the signature in PCa. The CLCs signature was developed based on the following formula: Riskscore = 0.790 × CLCN2 - 0.136 × CLCN6. (**Figures 2D, E**). We split PCa patients into the high-risk and low-risk groups with the median risk score as the threshold. Survival analysis indicated that the BCR-free time of the high risk PCa patients was markedly shorter than that of its counterpart (**Figure 3A**), which was validated in internal and external cohorts, namely the testing TCGA-PRAD cohort, the entire TCGA-PRAD cohort, and GSE21034 cohort (**Figures 3B-D**). ROC curves analysis were introduced to assess the efficiency of our signature in BCR-free survival prediction. The AUC value was 0.749, 0.674, and 0.754 at 1-, 2-, and 3- years in the training cohort, respectively (**Figure 3A**). The excellent sensitivity and specificity of our signature was verified in the other three internal and external cohorts (**Figures 3B-D**). Additionally, the distribution of risk scores, survival status, and heatmap in the training cohort were consistent with the results of the testing TCGA-PRAD, entire TCGA-PRAD, and GSE21034 cohorts (**Figures 4A-D**).

### Correlation between prognostic signature and clinical features

**Figure 5:**
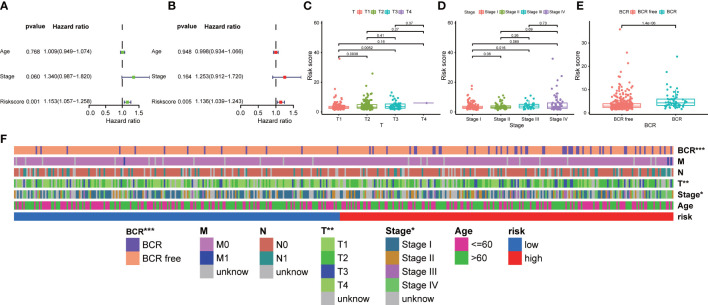
Correlation between prognostic signature and clinical features. **(A)** Univariate and **(B)** multivariate regression analyses of the signature in the training TCGA-PRAD cohort. The scatter diagrams indicated higher risk score meant advanced **(C)** T stage, **(D)** stage, and more **(E)** BCR events in the entire TCGA-PRAD cohort. **(F)** The band diagram of clinical features between the high- and low-risk groups in the entire TCGA-PRAD cohort. * p<0.05, ** p<0.01, and *** p<0.001.

In addition, we explored the correlation between our prognostic signature and multiple clinical features in the training TCGA-PRAD cohort through univariate and multivariate Cox regression analyses. The univariate Cox regression analysis revealed that the ability of our signature in prognostic prediction was independent (**Figure 5A**). After adjusting for the confounding variables, the following multivariate Cox regression analysis also showed our signature was a prognostic factor (**Figure 5B**). Scatter diagrams indicated that PCa patients with advanced stage were inclined to have higher risk score, which meant more BCR events (**Figures 5C–E**). Band diagram showed there were significant differences between two subgroups in T stage, stage, and BCR (**Figure 5F**).

### Distribution analysis, establishment of a nomogram, and external model comparison

**Figure 6:**
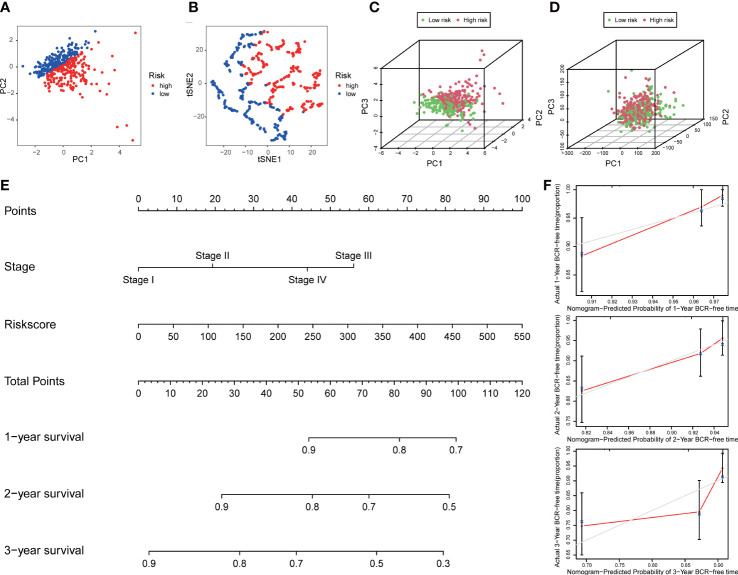
Distribution analysis and nomogram of the signature. **(A)** t-SNE and **(B)** 2D PCA analyses of the signature. 3D PCA analysis based on **(C)** all CLCs and **(D)** genome-wide expression profiles. **(E)** Construction of a nomogram predicted the probability of 1-, 2-, and 3- years BCR-free survival of PCa patients. **(F)** The calibration curves verified the accuracy of nomogram.

**Figure 7:**
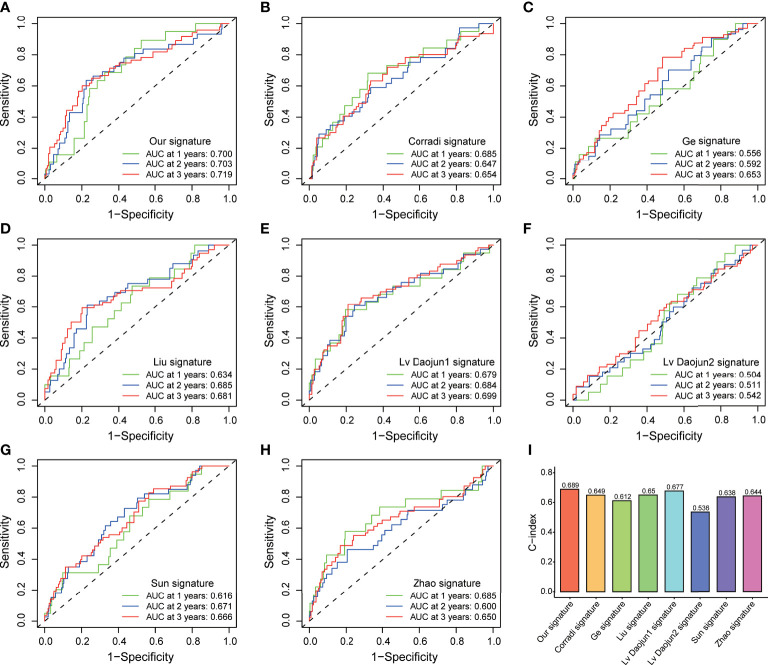
External model comparison analysis. **(A-H)** The ROC analysis curves and **(I)** C-index of the signature and multiple external models.

PCA and t-SNE analyses indicated that PCa patients in both subgroups were well stratified into two different distributed sections (**Figures 6A, B**), while the chaotic distributions were present in all CLCs and genome-wide expression (**Figures 6C, D**), indicating the excellent predictive ability of our prognostic signature. Furthermore, we constructed a nomogram to quantitatively predict the probability of 1-, 2-, and 3- years BCR-free survival of PCa patients (**Figure 6E**). Excellent agreements were reached on calibration curves of the prediction probability of BCR-free survival (1-, 2-, and 3-years) (**Figure 6F**). Compared with multiple external models, the C-index was higher than any other signatures, which further affirmed the accuracy and practicability of our signature (**Figures 7A-I**).

### Functional analyses

**Figure 8:**
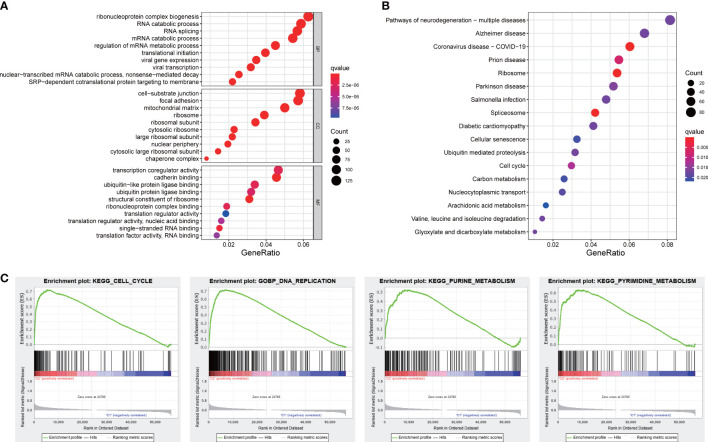
Functional analyses. **(A)** GO and **(B)** KEGG pathways enrichment analysis. **(C)** The GSEA analysis.

Functional analyses of DEGs between two subgroups were conducted to investigate related pathways and biological processes. GO analysis revealed that our signature was mainly involved in the assembly of genetic material in cell nucleus or mitochondria, such as mRNA catabolic process, regulation of mRNA metabolic process, and mitochondrial matrix (**Figure 8A**). The items of KEGG results were mostly enriched in cellular process and metabolic process, such as cell cycle, carbon metabolism, arachidonic acid metabolism, and glyoxylate and dicarboxylate metabolism (**Figure 8B**). Furthermore, the results of GSEA analysis also indicated our signature was primarily concentrated in cellular process and metabolic process, including cell cycle, DNA replication, and energy metabolism pathways (**Figure 8C**).

### The expression patterns of core genes and the connection to PCa progression

**Figure 9:**
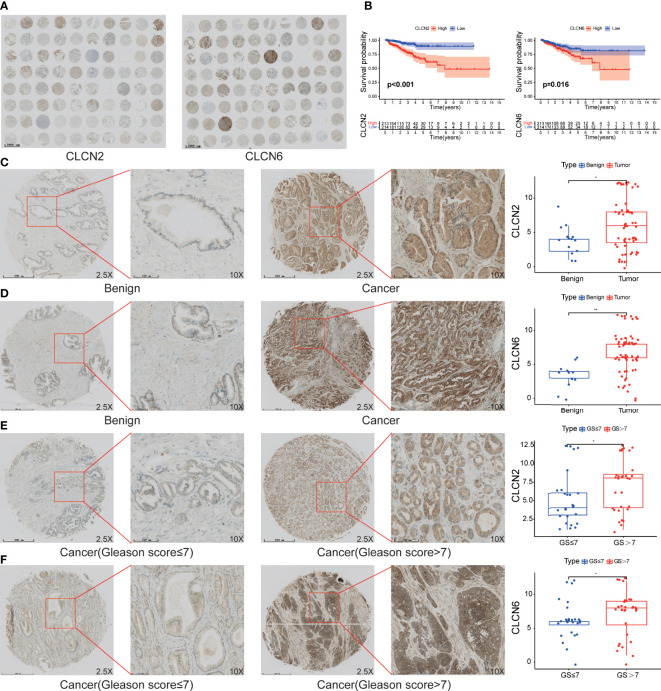
The expression patterns of core genes and the connection to PCa progression. **(A)** The overall view of CLCN2 and CLCN6 based on IHC analysis. **(B)** Kaplan-Meier survival of BCR based on CLCN2 and CLCN6 expression from TCGA database. IHC of TMA revealed **(C)** CLCN2 and **(D)** CLCN6 were upregulated in PCa. Greater **(E)** CLCN2 and **(F)** CLCN6 stainings were related to higher Gleason score (GS). * p<0.05 and ** p<0.01.

In order to investigate the expression patterns of CLCN2 and CLCN6 in PCa, we evaluated the differences between benign and PCa tissues in TMA based on IHC analysis. The overall view of CLCN2 and CLCN6 was depicted in **Figure 9A**. We observed that PCa patients with high CLCN2 or CLCN6 expression had worse BCR-free survival *via* Kaplan-Meier survival plots based on the optimal threshold in the TCGA-PRAD cohort (**Figure 9B**). Besides, the expression levels of CLCN2 and CLCN6 in PCa tissues was higher distinctly than that in benign prostate tissues (**Figures 9C, D** **;** **Table 1**, CLCN2, IRS: PCa = 6.05 ± 3.67 vs benign = 3.86 ± 2.14, *P*< 0.05; CLCN6, IRS: PCa = 6.77 ± 3.12 vs benign = 3.31 ± 1.84, *P*< 0.01). To evaluate the relationship between CLCN2 or CLCN6 expression levels and kinds of clinicopathological characteristics, statistically and more importantly, higher CLCN2 or CLCN6 expression in PCa tissues was positively correlated to high Gleason score (**Figures 9E, F** **;** **Table 1**, CLCN2, IRS: Gleason score > 7 = 7.03 ± 3.56 vs Gleason score ≤ 7 = 5.15 ± 3.46, *P*< 0.05; CLCN6, IRS: Gleason score > 7 = 7.13 ± 3.23 vs Gleason score ≤ 7 = 6.18 ± 2.76, *P*< 0.05).

### Downregulation of CLCN2 or CLCN6 suppressed cell proliferation and colony formation

**Figure 10:**
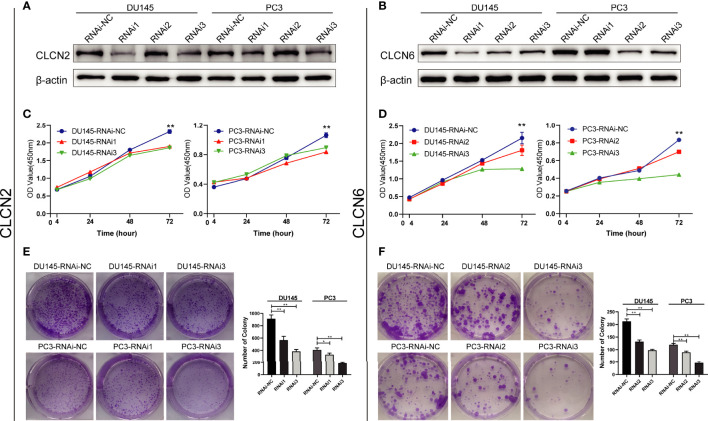
Downregulation of CLCN2 or CLCN6 inhibited cell proliferation and colony formation. WB assay of **(A)** CLCN2 or **(B)** CLCN6 downregulation efficiency. Downregulation of **(C, E)** CLCN2 or **(D, F)** CLCN6 suppressed cell proliferation and colony formation based on CCK-8 and colony formation analysis. * p<0.05 and ** p<0.01.

To explore the role of CLCN2 and CLCN6 expression in the progression of PCa, we established the model of downregulation of CLCN2 or CLCN6 owing to their upregulation in PCa tissues. The downregulation efficiency was verified by WB assay. The results showed CLCN2 or CLCN6 expression levels of the downregulation groups were lower than those of the control group of DU145 and PC3 cell lines (**Figures 10A, B**). To prevent off-target effects of siRNA, RNAi1 & RNAi3 of CLCN2 and RNAi2 & RNAi3 of CLCN6 were selected for next studies. Additionally, the CCK8 assay indicated that downregulation of CLCN2 or CLCN6 protein inhibited the cell proliferation of PCa (**Figures 10C, D**). The colony formation assay also revealed that the colony formation rates of CLCN2 or CLCN6 downregulation were significantly diminished compared to corresponding control group (**Figures 10E, F**).

### Downregulation of CLCN2 or CLCN6 inhibited migration, invasion, but prolonged cell cycle and promoted apoptosis

**Figure 11:**
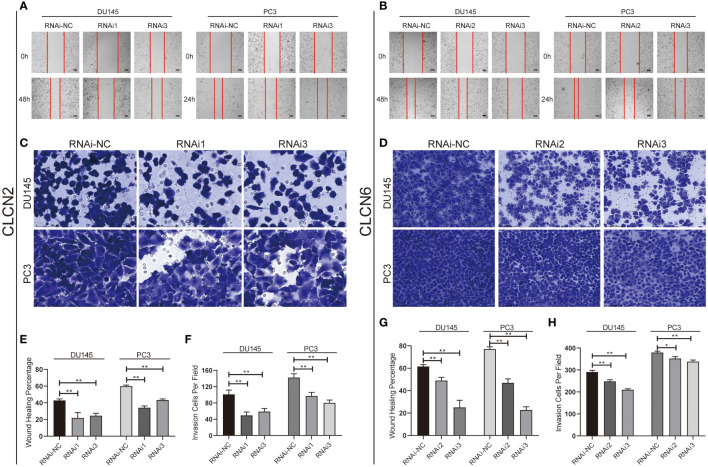
Downregulation of CLCN2 or CLCN6 inhibited cell migration and invasion. Downregulation of **(A, E)** CLCN2 or **(B, G)** CLCN6 inhibited migration. Downregulation of **(C, F)** CLCN2 or **(D, H)** CLCN6 inhibited invasion. * p<0.05 and ** p<0.01.

**Figure 12:**
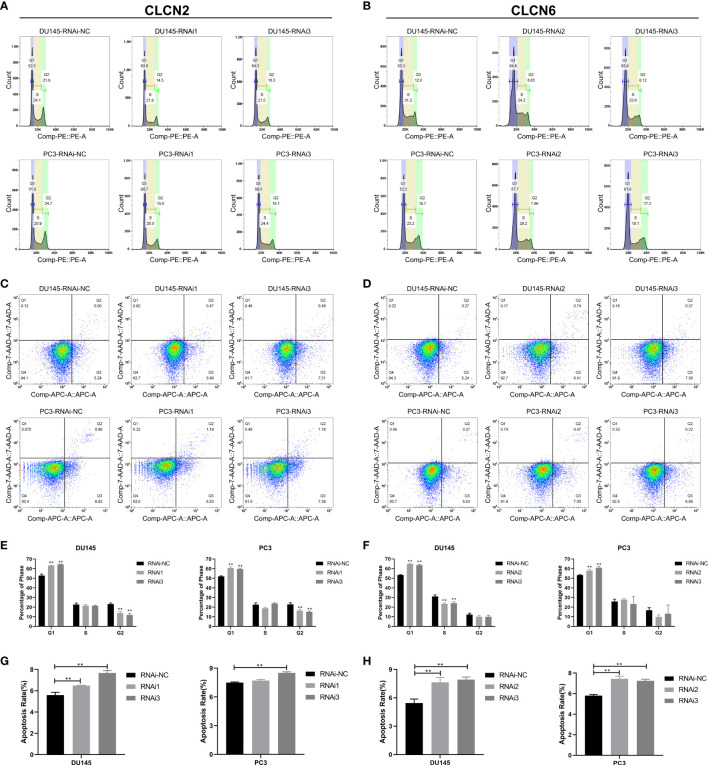
Downregulation of CLCN2 or CLCN6 prolonged cell cycle and promoted apoptosis. Downregulation of **(A, E)** CLCN2 or **(B, F)** CLCN6 could prolong cell cycle progression. Downregulation of **(C, G)** CLCN2 or **(D, H)** CLCN6 increased apoptosis. ** p<0.01.

Wound-healing assay and transwell assay were conducted to determine the relationship between CLCN2 or CLCN6 expression and PCa cell migration along with invasion. Our results indicated that downregulation of CLCN2 or CLCN6 extraordinarily suppressed both migration (**Figures 11A, B, E, G**) and invasion abilities (**Figures 11C, D, F, H**) of PCa cell lines compared to the corresponding control groups. Furthermore, we observed that downregulation of CLCN2 or CLCN6 could prolong the cell cycle progression by increasing the proportion of G1 phase cells (**Figures 12A, B, E, F**). On the contrary, downregulation of CLCN2 or CLCN6 in the DU145 and PC3 cell lines would dramatically increase apoptotic rates of PCa (**Figures 12C, D, G, H** **)**.

### Downregulation of CLCN2 or CLCN6 exerted an inhibitory effect on energy metabolism

**Figure 13:**
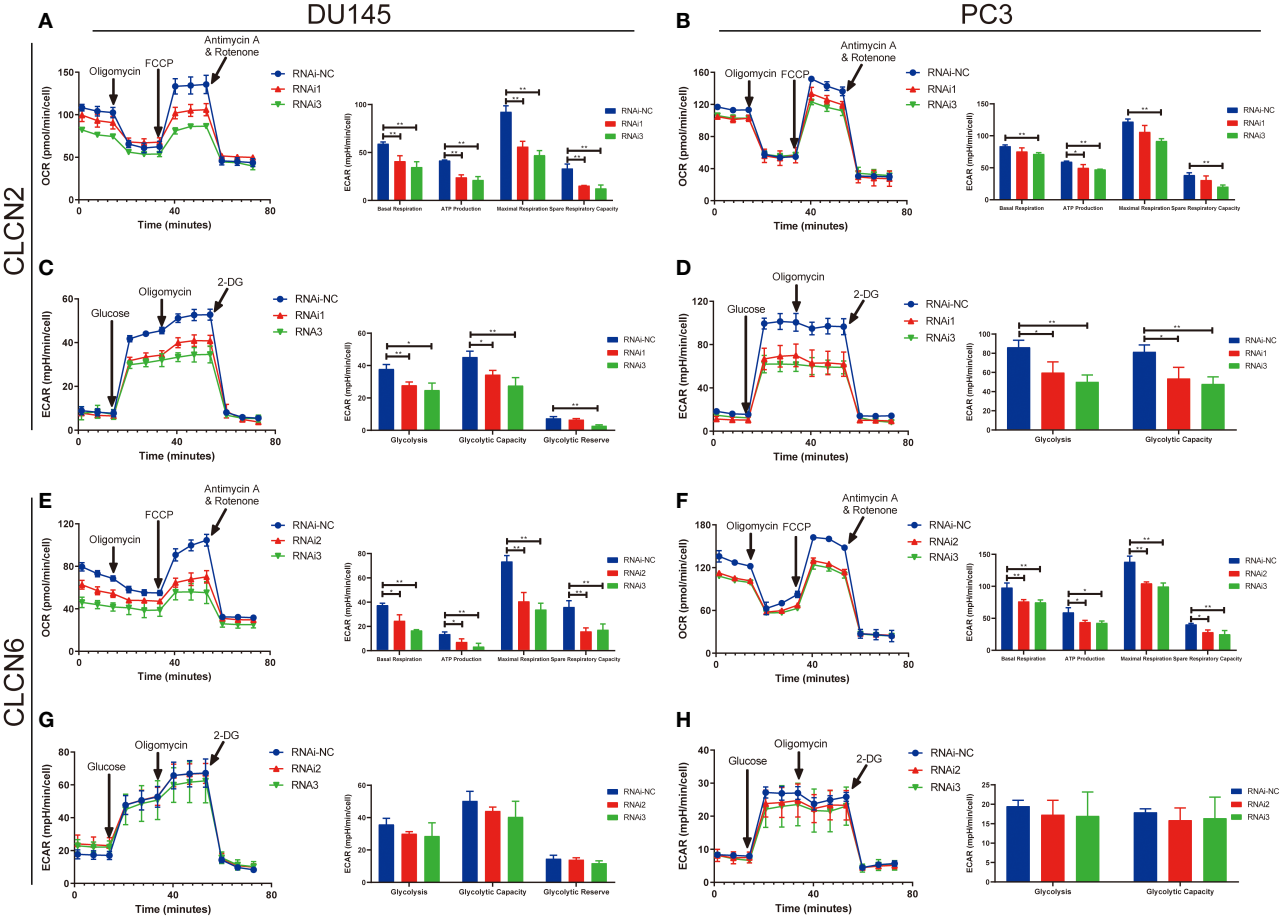
Downregulation of CLCN2 or CLCN6 exerted inhibitory effects on energy metabolism. Downregulation of **(A, B)** CLCN2 or **(E, F)** CLCN6 inhibited basal respiration, ATP production, maximal respiration, and spare respiratory capacity. Downregulation of **(C, D)** CLCN2 inhibited glycolysis, glycolytic capacity, and glycolytic reserve. **(G, H)** There was no significant difference when CLCN6 was downregulated. * p<0.05 and ** p<0.01.

The results of enrichment analysis indicated that our signature was closely related to metabolic process. Therefore, in the cause of studying the effects of CLCN2 and CLCN6 expression on metabolic process, the Seahorse XF96 extracellular flux analyzer was applied to explore how downregulation of CLCN2 or CLCN6 affected glycolytic function and mitochondrial function in PCa cells. Glycolytic function measuring the ECAR provided a standard and comprehensive method to assess the key parameters of glycolytic flux. Mitochondrial function by directly measuring the OCR to reveal the key parameters using the Seahorse XF96 cell mito stress test. In our study, we observed some pivotal parameters of basal respiration, ATP production, maximal respiration, and spare respiratory capacity were all reduced compared to the control group when CLCN2 or CLCN6 was downregulated (**Figures 13A, B, E, F**). Moreover, we found downregulation of CLCN2 greatly inhibited glycolysis, glycolytic capacity, and glycolytic reserve (**Figures 13C, D**), while there was no significant difference when CLCN6 was downregulated (**Figures 13G, H**).

## Discussion

Recent study indicated that PCa would become the first frequent cancer and the second tumor-related mortality among men in 2022 (29). More and more scientific researchers have attached importance to discover some effective diagnostic and prognostic indicators of PCa (30, 31). In our study, we constructed and validated a brand new signature comprising of CLCN2 and CLCN6 based on CLCs related genes that could predict the BCR of PCa, was an independent excellent prognostic factor, and was extraordinarily involved in cellular process and metabolic process. Compared with seven external models that using mRNAs to develop prognostic signature in PCa, the number of genes in our signature was smallest, which could be convenient for clinical practice and promotion, achieving higher clinical utility. Additionally, the highest C-index and the close connection to prognostic characteristics meant the excellent predictive accuracy of our signature, facilitating the early warning of tumor occurrence and progression in clinical practice. Therefore, our signature was more reliable for the prediction of PCa recurrence. Furthermore, we observed CLCN2 and CLCN6 expression were upregulated in PCa samples compared to benign prostate tissues. The analysis on the TMA of our cohort identified that upregulation of CLCN2 or CLCN6 was significantly related to higher Gleason score. PCa patients with higher CLCN2 or CLCN6 expression had a worse BCR-free survival. All above findings suggested the evidence that upregulation of CLCN2 or CLCN6 could lead to carcinogenesis, aggressive progression, and poor survival outcomes of PCa patients. Functionally, downregulation of CLCN2 or CLCN6 inhibited cell proliferation, colony formation, migration, invasion, but prolonged cell cycle, and promoted apoptosis. Additionally, downregulation of CLCN2 or CLCN6 exerted inhibitory effects on energy metabolism, which may be promising targets to treat PCa.

Ion channels and transporters were involved the effects in the proliferation, migration, and invasion of various cancers, indicating the physiological intracellular effects as potential targets for cancer treatments (32). Liu et al. determined LRRC8/VRAC, a volume-regulated anion channel, was greatly essential for cell proliferation and migration in glioblastoma cells (33). Wang et al. identified that simvastatin could inhibited the activity of Ca^2+^-activated chloride channel to repress cell proliferation in oral squamous cell carcinoma through TMEM16A (34).

CLCs were permeable channel proteins for chloride ions and other anions on the organism membrane. They could be activated under the condition of cell swelling, hyperpolarization, extracellular acidification, and extracelluar hypotonicity (35–38). Villaz et al. pointed out that both cell cycle and cell volume could modulate CLCs in ascidian embryos (39). Lepple-Wienhues et al. reported that the tyrosine kinase p56lck could alter lymphocytes swelling by chloride channels (40). An increase in cell volume promoted cell proliferation, while cell shrinkage triggered cell apoptosis according to various previous studies (7, 9). Enrichment analysis on cancer-related pathways and processes correlated to our signature indicated that alteration of CLCN2 or CLCN6 expression could affect cell volume based on biological regulation and assembly of genetic material. In addition, the results of various cell experiments were consistent with the findings that downregulation of CLCN2 or CLCN6 contributed to the prolongation of cell cycle and the deceleration of cell proliferation.

We were the first to explore the role of CLCs in the energy metabolism of PCa cells. Our results indicated that downregulation of CLCN2 or CLCN6 exerted an inhibitory effect on energy metabolism. Tumor cell metabolism had been proved to be greatly important to carcinogenesis and progression of cancer *via* providing sufficient energy (41). ATP was the most direct source of energy in organisms, most of which was obtained from mitochondrial metabolism. Mitochondria was a central hub for oxidative metabolism and responded to cellular stress, which affected many important cellular processes such as cell proliferation, invasion, apoptosis, and metastasis (23, 42, 43). Studies have revealed that CLCN2 and CLCN6 could participate in the regulation of various energy metabolism and other cancer biology. High glucose could decline the volume-activated Cl^-^ current and CLCN2 expression through suppressing PI3K signaling pathway, contributing to the decline of cell migration of keratinocytes (20). Another study revealed that intracellular ATP could alter the surface expression of CLCN2 (21). Additionally, CLCN2 has been found to predict the development and occurrence of lung cancer and might serve as a novel molecular therapeutic target of non-small cell lung cancer (44). Additional research evidence indicated that CLCN2 was significantly up-regulated and could function as a prognostic factor in hepatocellular carcinoma (45). Polymorphism at methylenetetrahydrofolate reductase CLCN6 was closely related to keratinocyte cancer in renal transplant recipients (46). In line with the above discussion, we found that the cancer-related pathways correlated to our signature were primarily enriched in cellular process and metabolic process. In the mito stress test of Seahorse assay, downregulation of CLCN2 or CLCN6 suppressed the mitochondrial function, including basal respiration, ATP production, maximal respiration, and spare respiratory capacity. These results indicated that downregulation of CLCN2 or CLCN6 could affect cell proliferation, invasion, and apoptosis by decreasing ATP production. The molecular potential mechanism needed further investigation. In addition, the glycolysis stress test of Seahorse assay was performed to detect the glycolytic function. PCa could exert a regulating activity on glycolysis based on androgen receptor signaling, thereby promoting tumor metabolism and growth (47). The overactivation of glycolysis was related to the poor prognosis of PCa. Chen et al. pointed out that FV-429 could exerted significant tumor-suppressive effect based on glycolysis inhibition and apoptosis by downregulating the AR-AKT-HK2 signaling axis (47). Furthermore, glucose transporter 1 was one of the most widely expressed transporter isoforms in tumors, which may play a significant role in PCa progression by regulating glycolysis and proliferation (48, 49). Consisting with our results, downregulation of CLCN2 repressed glycolysis, glycolytic capacity, and glycolytic reserve, which might inhibited PCa progression by mediating glycolysis and proliferation. Nevertheless, there was no significant difference in glycolysis analysis when CLCN6 was downregulated. The specific mechanism needed the next investigation. Thus, CLCN2 or CLCN6 may serve as promising therapeutic targets *via* mediating energy metabolism and proliferation for the treatment of PCa.

Nevertheless, there were still some limitations of our study. Firstly, our signature constructed by available public datasets should be validated in our own clinical cohort of PCa from the hospital. Secondly, a certain detailed systematic therapeutical information should be integrated into our signature to verify our conclusions. Finally, the underlying molecular mechanisms of our signature, especially how CLCN2 and CLCN6 regulated ion transport process, were required for the next investigation.

## Conclusion

To sum up, our signature could provide a novel and robust strategy for the prognostic evaluation and improve treatment decision making for PCa patients. CLCN2 and CLCN6 could merge as promising prognostic markers and be related to mitochondrial function and glycolytic function, revealing that they could be potential targets to improve PCa treatment strategies.

## Data availability statement

The datasets presented in this study can be found in online repositories. The names of the repository/repositories and accession number(s) can be found in the article/supplementary material.

## Ethics statement

The studies involving human participants were reviewed and approved by the Ethics Committee of the Second People’s Hospital of Foshan. The patients/participants provided their written informed consent to participate in this study.

## Author contributions

YL designed the study, analyzed the data, performed the experiments, and drafted the article. XPL and XL searched literature and supervised the study. QC, JL, WZ, and XPL provided funds for the research. All authors contributed to the article and approved the submitted version.

## Funding

This study was supported by Guangdong Medical Science and Technology Research Fund Project (B2022332, B2020127, B2022106) granted to QC and XPL, Foshan Science and Technology Innovation Project (2020001005794) granted to JL, National Natural Science Foundation of China (82072813, 81571427), Guangzhou Municipal Science and Technology Project (201803040001, 202201020346), and the Science and Technology Development Fund (FDCT) of Macau SAR (0031/2021/A) granted to WZ.

## Acknowledgments

We would like to acknowledge the TCGA and GEO databases.

## Conflict of interest

The authors declare that the research was conducted in the absence of any commercial or financial relationships that could be construed as a potential conflict of interest.

## Publisher’s note

All claims expressed in this article are solely those of the authors and do not necessarily represent those of their affiliated organizations, or those of the publisher, the editors and the reviewers. Any product that may be evaluated in this article, or claim that may be made by its manufacturer, is not guaranteed or endorsed by the publisher.

## References

[1] Saad F. Quality of life in men with prostate cancer. Lancet Oncol (2019) 20:325–6. doi: 10.1016/S1470-2045(18)30863-5 30713037

[2] Grossman DC Curry SJ Owens DK Bibbins-Domingo K Caughey AB Davidson KW. Screening for prostate cancer: US preventive services task force recommendation statement. JAMA (2018) 319:1901–13. doi: 10.1001/jama.2018.3710 29801017

[3] Briganti A Joniau S Gandaglia G Cozzarini C Sun M Tombal B. Patterns and predictors of early biochemical recurrence after radical prostatectomy and adjuvant radiation therapy in men with pT3N0 prostate cancer: Implications for multimodal therapies. Int J Radiat Oncol Biol Phys (2013) 87:960–7. doi: 10.1016/j.ijrobp.2013.09.015 24351411

[4] Simmons MN Stephenson AJ Klein EA. Natural history of biochemical recurrence after radical prostatectomy: Risk assessment for secondary therapy. Eur Urol (2017) 51:1175–84. doi: 10.1016/j.eururo.2007.01.015 17240528

[5] Gu L Frommel SC Oakes CC Simon R Grupp K Gerig CY. BAZ2A (TIP5) is involved in epigenetic alterations in prostate cancer and its overexpression predicts disease recurrence. Nat Genet (2015) 47:22–30. doi: 10.1038/ng.3165 25485837

[6] Xu B Mao J Wang L Zhu L Li H Wang W. ClC-3 chloride channels are essential for cell proliferation and cell cycle progression in nasopharyngeal carcinoma cells. Acta Bioch Bioph Sin (2010) 42:370–80. doi: 10.1093/abbs/gmq031 20539936

[7] Jentsch TJ Pusch M. CLC chloride channels and transporters: Structure, function, physiology, and disease. Physiol Rev (2018) 98:1493–590. doi: 10.1152/physrev.00047.2017 29845874

[8] Lang F Föller M Lang K Lang P Ritter M Vereninov A. Cell volume regulatory ion channels in cell proliferation and cell death. Method Enzymol (2007) 428:209–25. doi: 10.1016/S0076-6879(07)28011-5 17875419

[9] Stauber T Weinert S Jentsch TJ. Cell biology and physiology of CLC chloride channels and transporters. Compr Physiol (2012) 2:1701–44. doi: 10.1002/cphy.c110038 23723021

[10] Jentsch TJ. CLC chloride channels and transporters: From genes to protein structure, pathology and physiology. Crit Rev Biochem Mol (2008) 43:3–36. doi: 10.1080/10409230701829110 18307107

[11] Lang F Shumilina E Ritter M Gulbins E Vereninov A Huber SM. Ion channels and cell volume in regulation of cell proliferation and apoptotic cell death. Contrib Nephrol (2006) 152:142–60. doi: 10.1159/000096321 17065810

[12] Hanahan D Weinberg RA. Hallmarks of cancer: The next generation. Cell (2011) 144:646–74. doi: 10.1016/j.cell.2011.02.013 21376230

[13] Hanahan D Weinberg RA. The hallmarks of cancer. Cell (2000) 100:57–70. doi: 10.1016/s0092-8674(00)81683-9 10647931

[14] Lastraioli E Iorio J Arcangeli A. Ion channel expression as promising cancer biomarker. Biochim Biophys Acta (2015) 1848:2685–702. doi: 10.1016/j.bbamem.2014.12.016 25542783

[15] Mitsuda M Shiozaki A Kudou M Shimizu H Arita T Kosuga T. Functional analysis and clinical significance of chloride channel 2 expression in esophageal squamous cell carcinoma. Ann Surg Oncol (2021) 28:5384–97. doi: 10.1245/s10434-021-09659-8 33565032

[16] Ishiguro T Avila H Lin S Nakamura T Yamamoto M Boyd DD. Gene trapping identifies chloride channel 4 as a novel inducer of colon cancer cell migration, invasion and metastases. Brit J Cancer (2010) 102:774–82. doi: 10.1038/sj.bjc.6605536 PMC2837579 20087350

[17] Peng F Cai W Li J Li H. ClC-5 downregulation induces osteosarcoma cell apoptosis by promoting bax and tBid complex formation. Front Oncol (2020) 10:556908. doi: 10.3389/fonc.2020.556908 33614474 PMC7892965

[18] Xie J Ye J Cai Z Luo Y Zhu X Deng Y. GPD1 enhances the anticancer effects of metformin by synergistically increasing total cellular glycerol-3-Phosphate. Cancer Res (2020) 80:2150–62. doi: 10.1158/0008-5472.CAN-19-2852 32179514

[19] Liu J Chen G Liu Z Liu S Cai Z You P. Aberrant FGFR tyrosine kinase signaling enhances the warburg effect by reprogramming LDH isoform expression and activity in prostate cancer. Cancer Res (2018) 78:4459–70. doi: 10.1158/0008-5472.CAN-17-3226 PMC6095720 29891507

[20] Pan F Guo R Cheng W Chai L Wang W Cao C. High glucose inhibits ClC-2 chloride channels and attenuates cell migration of rat keratinocytes. Drug Design Dev Ther (2015) 9:4779–91. doi: 10.2147/DDDT.S84628 PMC4560522 26355894

[21] Stölting G Teodorescu G Begemann B Schubert J Nabbout R Toliat MR. Regulation of ClC-2 gating by intracellular ATP. Pflugers Archiv: Eur J Physiol (2013) 465:1423–37. doi: 10.1007/s00424-013-1286-0 PMC3778897 23632988

[22] Jonckheere AI Smeitink JAM Rodenburg RJT. Mitochondrial ATP synthase: Architecture, function and pathology. J Inherit Metab Dis (2012) 35:211–25. doi: 10.1007/s10545-011-9382-9 PMC3278611 21874297

[23] Ahn CS Metallo CM. Mitochondria as biosynthetic factories for cancer proliferation. Cancer Metab (2015) 3:1. doi: 10.1186/s40170-015-0128-2 25621173 PMC4305394

[24] Pedersen PL. Mitochondria in relation to cancer metastasis: Introduction to a mini-review series. J Bioenerg Biomembr (2012) 44:615–7. doi: 10.1007/s10863-012-9470-z 22926290

[25] Luo Y Zhang G. Identification of a necroptosis-related prognostic index and associated regulatory axis in kidney renal clear cell carcinoma. Int J Gen Med (2022) 15:5407–23. doi: 10.2147/IJGM.S367173 PMC9173730 35685693

[26] Zhu H Wang N Yao L Chen Q Zhang R Qian J. Moderate UV exposure enhances learning and memory by promoting a novel glutamate biosynthetic pathway in the brain. Cell (2018) 173:1716–27. doi: 10.1016/j.cell.2018.04.014 29779945

[27] Liu Z Han Z Liang Y Chen J Wan S Zhuo Y. TRIB1 induces macrophages to M2 phenotype by inhibiting IKB-zeta in prostate cancer. Cell Signal (2019) 59:152–62. doi: 10.1016/j.cellsig.2019.03.017 30926388

[28] Liu R Feng Y Deng Y Zou Z Ye J Cai Z. A HIF1α-GPD1 feedforward loop inhibits the progression of renal clear cell carcinoma *via* mitochondrial function and lipid metabolism. J Exp Clin Cancer Res: CR (2021) 40:188. doi: 10.1186/s13046-021-01996-6 34098990 PMC8185942

[29] Siegel RL Miller KD Fuchs HE Jemal A. Cancer statistics, 2022. CA: Cancer J Clin (2022) 72:7–33. doi: 10.3322/caac.21708 35020204

[30] Lamy P Allory Y Gauchez A Asselain B Beuzeboc P de Cremoux P. Prognostic biomarkers used for localised prostate cancer management: A systematic review. Eur Urol Focus (2018) 4:790–803. doi: 10.1016/j.euf.2017.02.017 28753865

[31] Kretschmer A Tilki D. Biomarkers in prostate cancer - current clinical utility and future perspectives. Crit Rev Oncol/Hematol (2017) 120:180–93. doi: 10.1016/j.critrevonc.2017.11.007 29198331

[32] Shiozaki A Ichikawa D Otsuji E Marunaka Y. Cellular physiological approach for treatment of gastric cancer. World J Gastroentero (2014) 20:11560–6. doi: 10.3748/wjg.v20.i33.11560 PMC4155349 25206263

[33] Liu T Stauber T. The volume-regulated anion channel LRRC8/VRAC is dispensable for cell proliferation and migration. Int J Mol Sci (2019) 20:2663. doi: 10.3390/ijms20112663 PMC6600467 31151189

[34] Wang H Wang T Zhang Z Fan Y Zhang L Gao K. Simvastatin inhibits oral squamous cell carcinoma by targeting TMEM16A Ca(2+)-activated chloride channel. J Cancer Res Clin (2021) 147:1699–711. doi: 10.1007/s00432-021-03575-w PMC11801952 33755783

[35] Jordt SE Jentsch TJ. Molecular dissection of gating in the ClC-2 chloride channel. EMBO J (1997) 16:1582–92. doi: 10.1093/emboj/16.7.1582 PMC1169762 9130703

[36] Thiemann A Gründer S Pusch M Jentsch TJ. A chloride channel widely expressed in epithelial and non-epithelial cells. Nature (1992) 356:57–60. doi: 10.1038/356057a0 1311421

[37] Jentsch TJ Stein V Weinreich F Zdebik AA. Molecular structure and physiological function of chloride channels. Physiol Rev (2002) 82:503–68. doi: 10.1152/physrev.00029.2001 11917096

[38] Gründer S Thiemann A Pusch M Jentsch TJ. Regions involved in the opening of CIC-2 chloride channel by voltage and cell volume. Nature (1992) 360:759–62. doi: 10.1038/360759a0 1334533

[39] Villaz M Cinniger JC Moody WJ. A voltage-gated chloride channel in ascidian embryos modulated by both the cell cycle clock and cell volume. J Physiol (1995) 488(Pt 3):689–99. doi: 10.1113/jphysiol.1995.sp021000 PMC1156734 8576858

[40] Lepple-Wienhues A Szabò I Laun T Kaba NK Gulbins E Lang F. The tyrosine kinase p56lck mediates activation of swelling-induced chloride channels in lymphocytes. J Cell Biol (1998) 141:281–6. doi: 10.1083/jcb.141.1.281 PMC2132720 9531565

[41] Porporato PE Filigheddu N Pedro JMB Kroemer G Galluzzi L. Mitochondrial metabolism and cancer. Cell Res (2018) 28:265–80. doi: 10.1038/cr.2017.155 PMC5835768 29219147

[42] Bhandary B Marahatta A Kim H Chae H. Mitochondria in relation to cancer metastasis. J Bioenerg Biomembr (2012) 44:623–7. doi: 10.1007/s10863-012-9464-x 22914881

[43] Rahman KM Aranha O Glazyrin A Chinni SR Sarkar FH. Translocation of bax to mitochondria induces apoptotic cell death in indole-3-carbinol (I3C) treated breast cancer cells. Oncogene (2000) 19:5764–71. doi: 10.1038/sj.onc.1203959 11126363

[44] Xu Z Zheng X Zhao W Xin H Han Z. Expression and significance of chloride channel ClC-2 in nonsmall-cell lung cancer. Chin J Lab Diagn (2012) 16:60–2.

[45] Lu S Dai M Hu X Yi H Zhang Y. A new survival model based on ion channel genes for prognostic prediction in hepatocellular carcinoma. Genomics (2021) 113:171–82. doi: 10.1016/j.ygeno.2020.12.028 33340691

[46] Griffin L Ho L Akhurst RJ Arron ST Boggs JME Conlon P. Genetic polymorphism in methylenetetrahydrofolate reductase chloride transport protein 6 (MTHFR CLCN6) gene is associated with keratinocyte skin cancer in a cohort of renal transplant recipients. Skin Health Dis (2022) 2:e95. doi: 10.1002/ski2.95 35677930 PMC9168012

[47] Chen X Wei L Yang L Guo W Guo Q Zhou Y. Glycolysis inhibition and apoptosis induction in human prostate cancer cells by FV-429-mediated regulation of AR-AKT-HK2 signaling network. Food Chem Toxicol (2020) 143:111517. doi: 10.1016/j.fct.2020.111517 32619556

[48] Xiao H Wang J Yan W Cui Y Chen Z Gao X. GLUT1 regulates cell glycolysis and proliferation in prostate cancer. Prostate (2018) 78:86–94. doi: 10.1002/pros.23448 29105798

[49] Wang J Xu W Wang B Lin G Wei Y Abudurexiti M. GLUT1 is an AR target contributing to tumor growth and glycolysis in castration-resistant and enzalutamide-resistant prostate cancers. Cancer Lett (2020) 485:45–55. doi: 10.1016/j.canlet.2020.05.007 32428663

